# Modified silicone stent for the treatment of post-surgical bronchopleural fistula: a clinical observation of 17 cases

**DOI:** 10.1186/s12890-020-01372-8

**Published:** 2021-01-06

**Authors:** Junli Zeng, Xuemei Wu, Zhide Chen, Meihua Zhang, Mingyao Ke

**Affiliations:** Department of Respiratory Centre, The Second Affiliated Hospital of Xiamen Medical College, 566 Shengguang Road, Xiamen, 361000 Fujian Province China

**Keywords:** Modified silicone stent, Bronchopleural fistula, Rigid bronchoscopy

## Abstract

**Background:**

Bronchopleural fistula is a rare but life-threatening event with limited therapeutic options. We aimed to investigate the efficacy and safety of the modified silicone stent in patients with post-surgical bronchopleural fistula.

**Methods:**

Between March 2016 and April 2020, we retrospectively reviewed the records of 17 patients with bronchopleural fistula and who underwent bronchoscopic placement of the Y-shaped silicone stent. The rate of initial success, clinical success and clinical cure, and complications were analyzed.

**Results:**

Stent placement was successful in 16 patients in the first attempt (initial success rate: 94.1%). The median follow-up time was 107 (range, 5–431) days. All patients achieved amelioration of respiratory symptoms. The clinical success rate was 76.5%. Of the 14 patients with empyema, the daily drainage was progressively decreased in 11 patients, and empyema completely disappeared in six patients. Seven stents were removed during follow-up: four (26.7%) for the cure of fistula, two for severe proliferation of granulomatous tissue and one for stent dislocation. No severe adverse events (i.e. massive hemoptysis, suture dehiscence) took place. Seven patients died (due to progression of malignancy, uncontrolled infection, myocardial infarction and left heart failure).

**Conclusions:**

The modified silicone stent may be an effective and safe option for patients with post-surgical bronchopleural fistula patients in whom conventional therapy is contraindicated.

## Background

Bronchopleural fistula (BPF), the aberrant connection between the bronchial tree and the pleural cavity, is a severe complication of pulmonary resection surgery. Despite the advances in surgical techniques [1, 2], BPF still occurred in 4.5–20% of patients after pneumonectomy, and in 0.5–15% of patients after lobectomy [3, 4]. The mortality rate could be as high as 71% [5].

No consensus on the best treatment of BPF has been established. Therapeutic options range from extensive surgical procedures to bronchoscopic techniques. The surgical treatments include open thoracostomy, thoracoplasty, and direct closure of the fistula with flaps of different origins [6]. However, surgery cannot be readily tolerated in patients with a poor overall condition. Moreover, the recurrence rate of BPF after surgical repair could be as high as 23.6% [6]. The recurrence rate of bronchopleural fistula after surgical repair carries a mortality exceeding 50%, largely due to respiratory insufficiency and uncontrolled sepsis [7].

Numerous bronchoscopic techniques have also been applied for closing the fistula. Airway stent placement plays an important role in the treatment of airway fistula [8]. The silicone stents have been extensively used in benign and malignant airway stenosis [9–11] but rarely applied in patients with airway fistula. The relatively poor adaptability might have limited further clinical application. However, silicone stents have some merits that may contribute to the success of fistula closure. Over the past 2 decades, the use of modified silicone stent in BPF has been reported in some case reports [12–14]. A case report has reported the use of the silicone stent placement for sealing the fistula, but the details of the operation have not been well elucidated.

Hence, we aimed to better address these limitations by applying the modified silicone stents in BPF patients. Based on the literature reports and our clinical experience, we sought to explore the feasibility, efficacy and safety of the modified silicone stent placement for BPF.

## Methods

### Patients and data review

In this retrospective study, we reviewed the medical records of all patients who had been treated with BPF through placement of the modified silicone stent between March 2016 and April 2020 in our study center. The protocol has been approved by the Ethics committee of The Second Affiliated Hospital of Xiamen Medical College and all patients signed written informed consent before performing rigid bronchoscopy.

The patients who met the following criteria underwent placement of the modified silicone stents. The inclusion criteria consisted of: (1) computed tomography (CT) and/or bronchoscopy-confirmed bronchopleural fistula; (2) the largest diameter of fistula being 3 mm or greater, or had minor fistula (diameter being 3 mm or less) which failed to be cured by conservative treatment (i.e. pleural cavity drainage, systematic antibiotics, and intrapleural antibiotic irrigation) and bronchoscopic sealants (i.e. fibrin glue, absolute ethanol injection, silver nitrate, coils); (3) surgical repair (i.e. resection, reconstruction) being technically inappropriate or contraindicated; (4) surgical failure or recurrence following surgery. We excluded the patients with the following conditions [15]: (1) hemodynamic instability; (2) severe cardiovascular and cerebrovascular diseases; (3) severe thrombocytopenia and/or severe coagulopathy; (4) cervical spine instability; (5) severe facial injuries that hampered the placement of rigid bronchoscopy.

We manually searched the medical records and extracted the clinical information and bronchoscopic data. Initial success was defined as the successful insertion of the stent, which immediately ceased the air leak from the residual cavity after stenting. Clinical success was defined as a major relief of symptoms, no air leakage, no sign of persistent fistula, and a notably reduced volume of daily drainage lasting for more than 1 month.

### The bronchoscopic procedure and fabrication of silicone stent

**Fig. 1:**
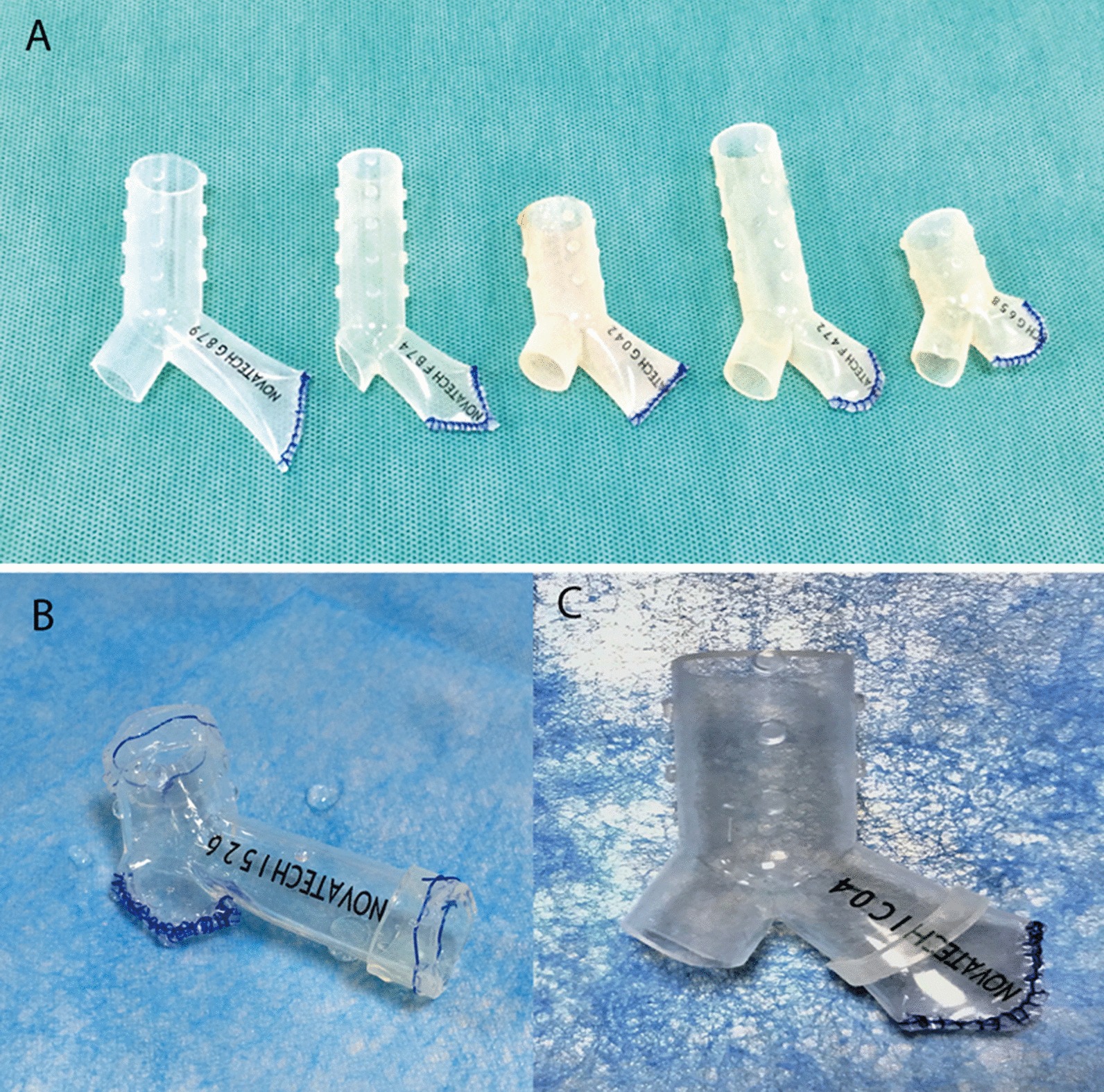
Illustration of the modified silicone stent. **a** The modified silicone stent consists of the main branch, the lateral branch and the occluded branch; **b** the stent rings are sutured to the main branch and the lateral branch of silicone stent; **c** the stent ring is sutured to the occluded branch

The flexible bronchoscopy was performed before stent placement to identify the site of fistula orifice and clear away the secretion. For the fistula which could not be visualized by bronchoscopy, a balloon occlusion test or instillation of methylene blue would be performed. The site and size of the fistula were measured by multi-slice CT (with three-dimensional reconstructions) and bronchoscopy. We use the sterilized straight stent segments with different diameter as a measurement tool to determine the optimal size of the stent. According to the measurement, the silicone stent (TRACHEBRONXANE™ DUMON®, Novatech, France) with the optimal diameter was chosen. The selected stent was then modified on site to fit the individual airways. The modified stent was fabricated manually based on the Y-shaped silicone stent by tailoring and suturing. The finished stent composed of the occluded branch, the main branch and the lateral branch (Fig. 1a). The suitable stent ring was nested or sutured to the selected stent if the original size of the stent was not fit (Fig. 1b, c). Next, the modified Dumon stent was placed into the involved bronchus through the rigid bronchoscope. Details of the procedure have been published previously [16]. Further information about the process of modifying and placing the stent is available in Additional file 1: The procedure of modifying and placing the silicone stent.

Finally, the modified Dumon stent was placed and the involved bronchus (lobar bronchus or main bronchus) was excluded according to the site of fistula. Because of the corresponding lobe of the bronchus that we have excluded were resected before stent placement in most of our cases, the operation would hardly result in a major decrease in the lung function. All patients were carefully and continuously monitored. To confirm the location of stent and the patency of airway, all patients were requested to undergo bronchoscopy and chest CT within a week.

### Other management

In case of early BPF and BPF with empyema, an urgent pleural drainage would be required. The intercostal tube drainage with or without suction was used. All patients received antibiotic therapy adjusted according to bacterial culture findings. Irrigation of the pleural would be needed in case empyema occurred. Nebulization treatment was empirically applied to improve the retention of phlegm. The chest tube could be removed if there was no air leakage, no sign of persistent fistula, the daily volume of drainage was less than 20 ml/h or the purulent fluid disappeared.

### Follow-up

After stenting, the patients were followed up without a uniform schedule. The frequency depended upon each patient’s general well-being and airway’s conditions. The airway condition was checked monthly by bronchoscopy. The shrinkage of the thoracic cavity was followed up monthly with chest CT. More frequent test would be needed if the patients became symptomatic. The indications for clinical assessments included the development of respiratory symptoms, air leakage, an increase in the volume of daily drainage, a greater size of residual pleural cavity and the development of stent-related complications.

During follow-up, the stent would be removed in case of the following conditions: (1) the fistula being healed (closure of fistula that was confirmed by instillation of methylene blue and the drainage tube was removed with the disappearance of drainage fluid); (2) severe dislocation of the stent which was difficult to be adjusted by surgical grasping forceps or foreign-body forceps; (3) severe proliferation of granulomatous tissues that caused difficulty in breathing; (4) placement of stent not being tolerated by the patient.

### Statistical analysis

SPSS version 18.0 (SPSS Inc., USA) was used for data analysis. Continuous variables with normal distribution were expressed as mean ± standard deviations (normality of distribution verified by the Kolmogorov–Smirnov test); otherwise, the median and range would be reported. The change in variables over time was analyzed by paired t-test for data with normal distribution or non-parametric test for data without normal distribution. Categorical variables were presented as the absolute count (percentage).

## Results

A total of 17 patients with BPF (15 males and 2 females) who underwent placement of the modified stent were included. The mean age was 57.7 (range, 47–72) years. Fifteen patients had lung cancer and two had tuberculosis. Eight patients underwent lobectomy and nine underwent pneumonectomy. Bronchial stumps were closed by mechanical stapler in 11 patients. The median time from lung resection to the diagnosis of BPF was 42 days (range, 5 days–37 months). The median estimated diameter of fistula was 9.1 (range, 6–15) mm. Empyema was diagnosed in 14 patients, and the median volume of daily drainage was 116 (range, 20–270) ml. The detailed clinical characteristics are shown in Table 1.

**Table 1:** Clinical characteristics of patients

No	Age (years)	Primary disease	Surgical method	Empyema	Site of fistula
1	52	Tuberculosis	Left pneumonectomy	Yes	LMB
2	47	Adenocarcinomas	Left pneumonectomy	Yes	LMB
3	64	Adenocarcinomas	Right pneumonectomy	Yes	RMB
4	41	Squamous cell carcinomas	Left pneumonectomy	Yes	LMB
5	72	Squamous cell carcinomas	Left pneumonectomy	Yes	LSB
6	63	Squamous cell carcinomas	Right inferior lobectomy	No	RIB
7	59	Adenocarcinomas	Left inferior lobectomy	No	LIB
8	52	Tuberculosis	Right superior lobectomy	Yes	RSB
9	60	Squamous cell carcinomas	Right inferior lobectomy	Yes	RIB
10	47	Squamous cell carcinomas	Right inferior lobectomy	Yes	RIB
11	54	Squamous cell carcinomas	Right pneumonectomy	Yes	RMB
12	66	Squamous cell carcinomas	Right pneumonectomy	Yes	RMB
13	56	Squamous cell carcinomas	Right pneumonectomy	Yes	RMB
14	64	Adenocarcinomas	Left inferior lobectomy	Yes	LIB
15	62	Adenocarcinomas	Right inferior lobectomy	Yes	RIB
16	57	Squamous cell carcinomas	Right superior lobectomy	No	RSB
17	65	Squamous cell carcinomas	Right pneumonectomy	Yes	RMB

*RMB* right main bronchus, *LMB* left main bronchus, *LSB* left superior lobar bronchus; *RSB* right superior lobar bronchus, *RIB* right inferior lobar bronchus, *LIB* left inferior lobar bronchus

**Fig. 2:**
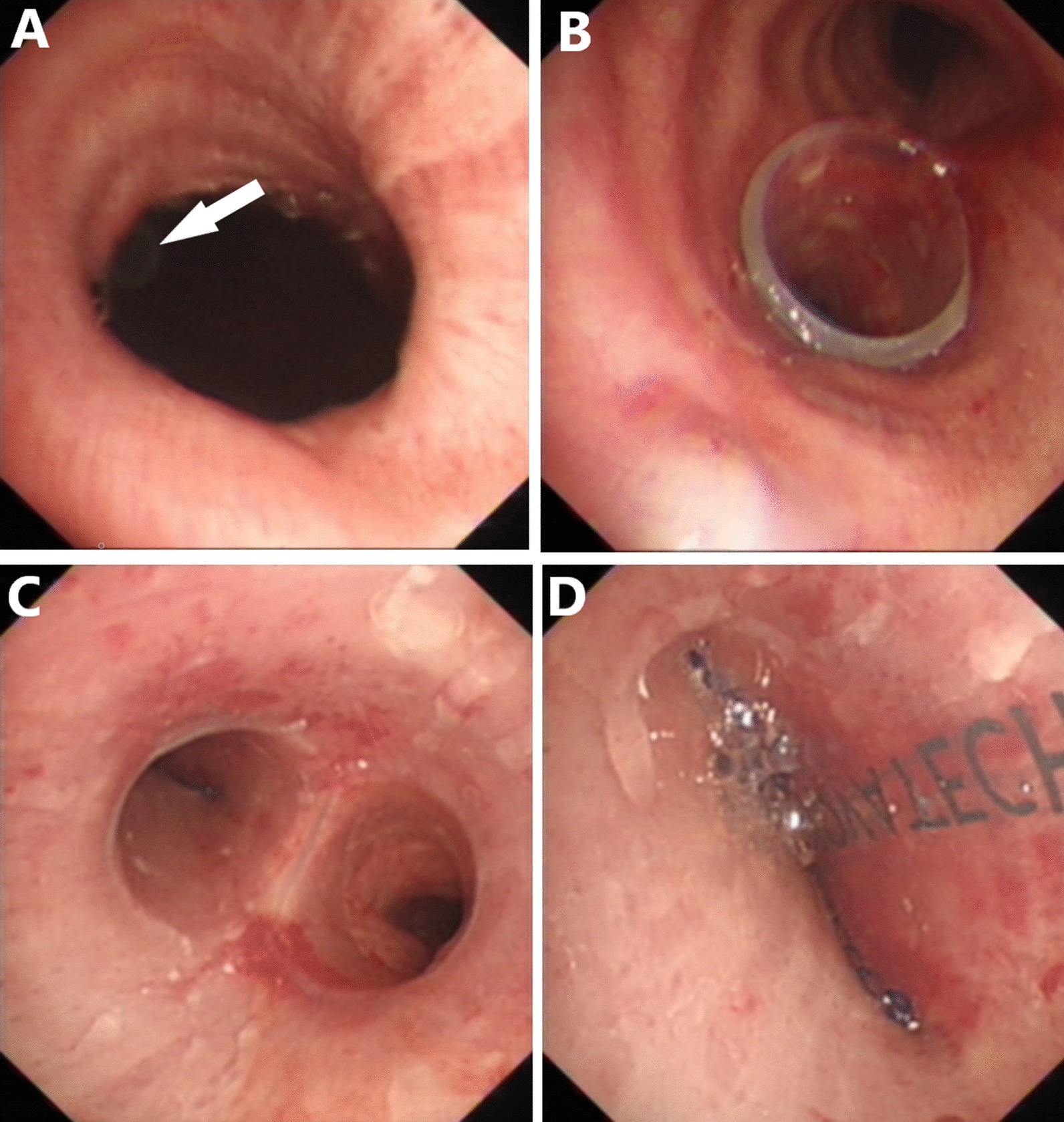
The bronchoscopic view of the stent placement in a representative case. Patient 5 is a 72-year-old male with BPF. **a** Bronchoscopic image of fistula in the left main bronchus and the drainage tube could be seen from the fistula (arrow); **b** the straight stent segment is used to measure the diameter of the left main bronchus; **c** bronchoscopic image of the carina, left main bronchus and right main bronchus after stenting; **d** the inner surface of the occluded branch

**Fig. 3:**
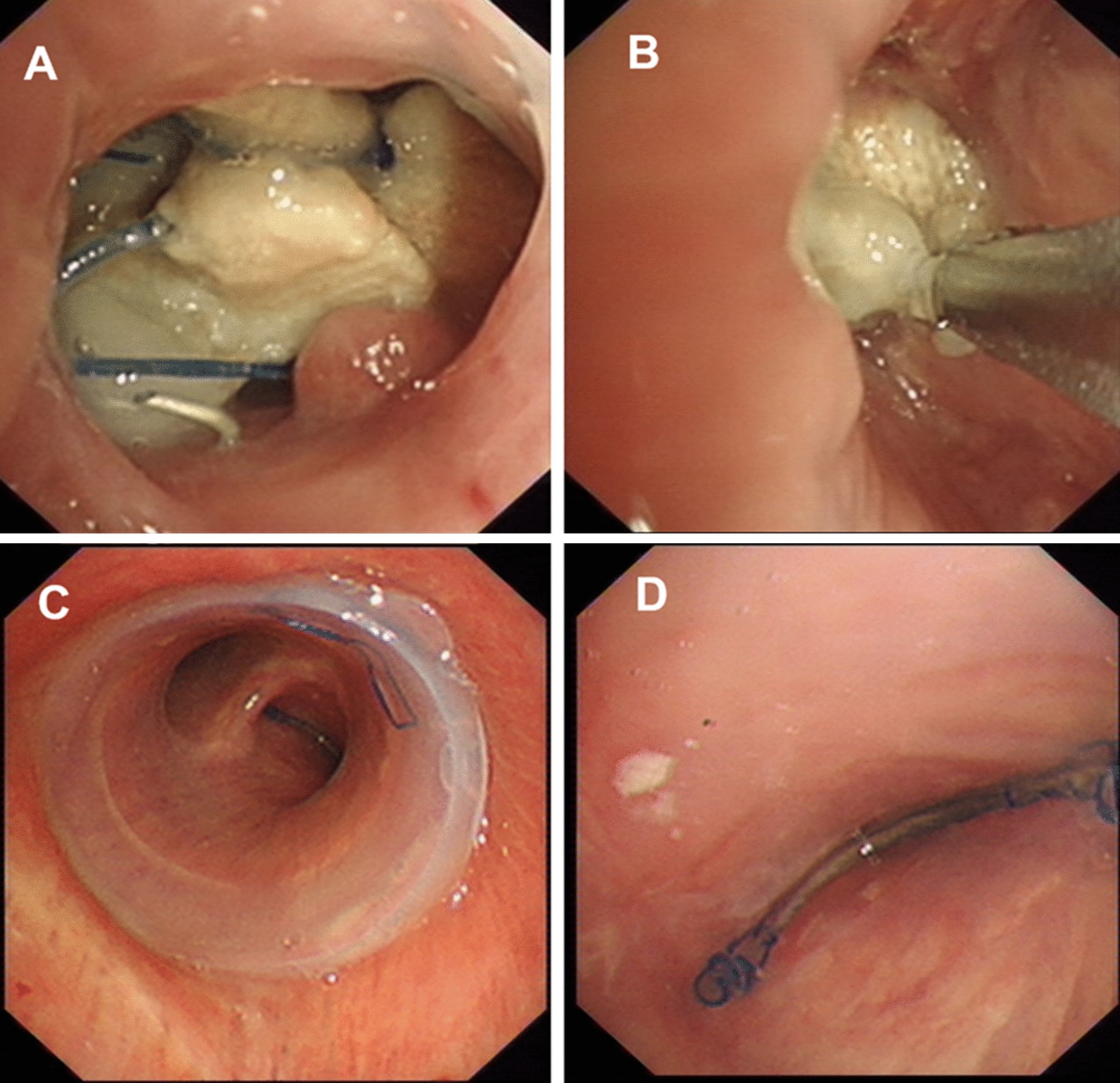
The bronchoscopic view of the stent placement in a representative case. Patient 11 is a 54-year-old male with BPF. **a** Images of the fistula orifice, from which the large amount of purulent secretions and surgical suture can be seen; **b** elimination of the purulent secretions under rigid bronchoscopy; **c** bronchoscopic image of the tracheal and carina after stenting; **d** the inner surface of the occluded branch in the right main bronchus

The modified silicone stents were successfully placed into the culprit bronchus in all patients. The modified stents were placed in conjunction with rings in four patients. The information of the stent type and the location of the occluded branch is described in the online Additional file 2: The type of the stents. Immediate cessation of air leakage was achieved in 16 patients (initial success rate: 94.1%). Only one patient failed to achieve an initial success due to the failure of matching between the occluded branch and the airway. The original stent was removed and fabricated by connecting a stent ring to the occluded branch. The new stent with a larger diameter was inserted. After the second operation, the fistula was closed and no gas spillover was observed in the chest draining tube. The bronchoscopic views of stent placement are shown in Figs. 2 and 3.

The median follow-up duration was 107 (range: 5–431) days among all patients. All patients reported amelioration of respiratory symptoms. The 14 patients who had empyema were treated with intercostal tube drainage and irrigation. One month after stenting, the volume of daily drainage was significantly reduced in 11 patients [median: 95 (range: 20–260) ml before and 20 (range: 0–70) ml after drainage, *P* = 0.001). Three patients did not attend follow-up. The median daily drainage volume at the end of follow-up was further decreased to 7.5 (range 0–20) ml. Overall, clinical success was achieved in 13 (76.5%) patients. Among the four patients who did not achieve clinical success, one suffered from recurrence of air leakage for the enlarged fistula at 15 days after stenting and three were lost to follow-up within 1 month.

**Fig. 4:**
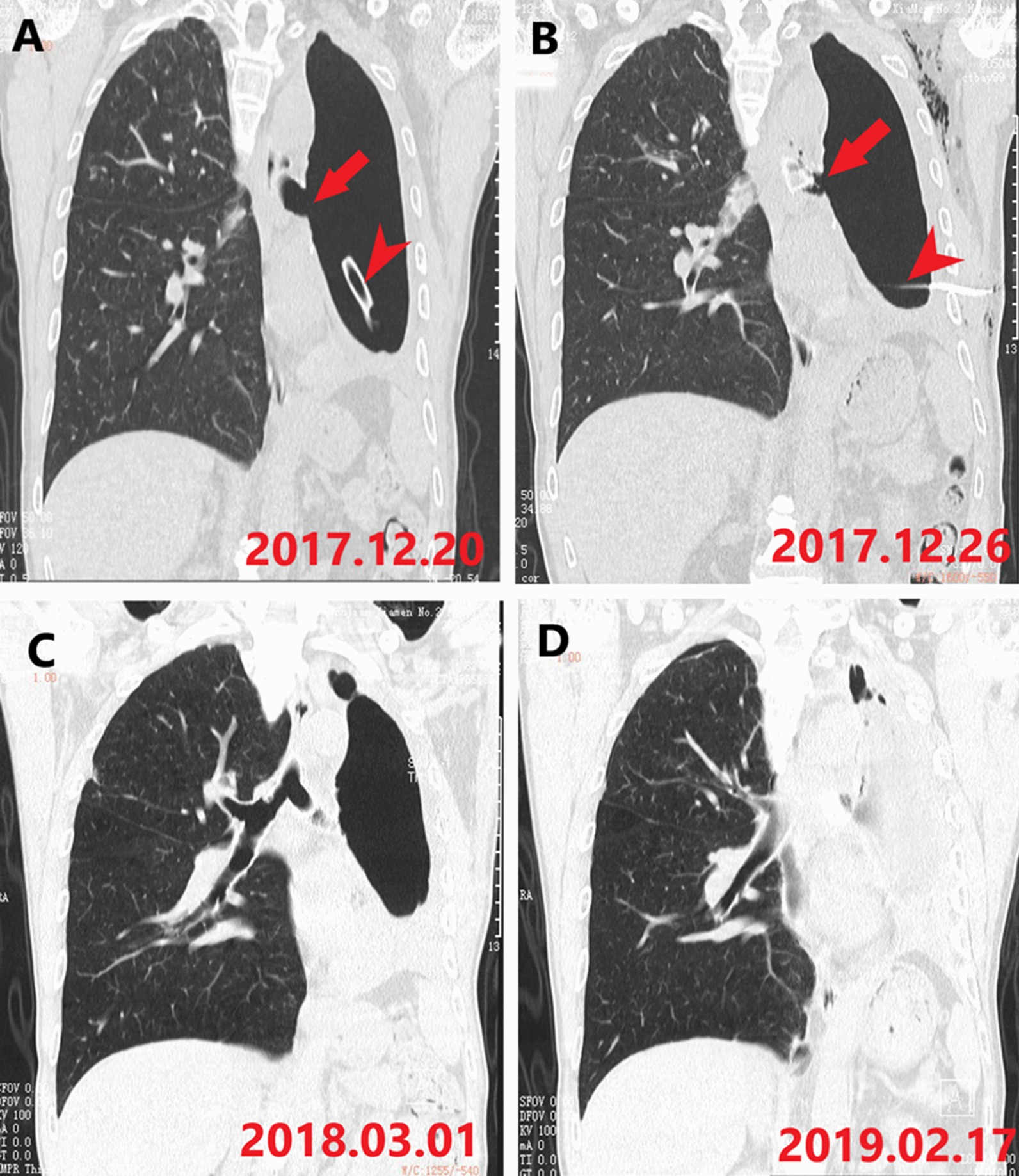
The coronal computer tomography image of patient 5 in different time. **a** The fistula in the left main bronchus (arrow) and the drainage tube (arrow head) is placed in the left thoracic cavity; **b** the occluded branch of the stent can be seen in the left main bronchus (arrow) and the drainage tube (arrow head) is still in the left thoracic cavity; **c** the residual pleural space diminishes over time; **d** the residual pleural space disappears during follow-up

At the end of follow-up, the drainage tube was removed in eight patients. The residual cavity has been progressively decreasing in 10 patients, and completely disappeared in six patients. The typical CT image is shown in Fig. 4. The stents were removed in seven patients. Four stents were removed when the fistula was cured—two for the severe granulomatous proliferation and one for the dislocation of the stent.

**Fig. 5:**
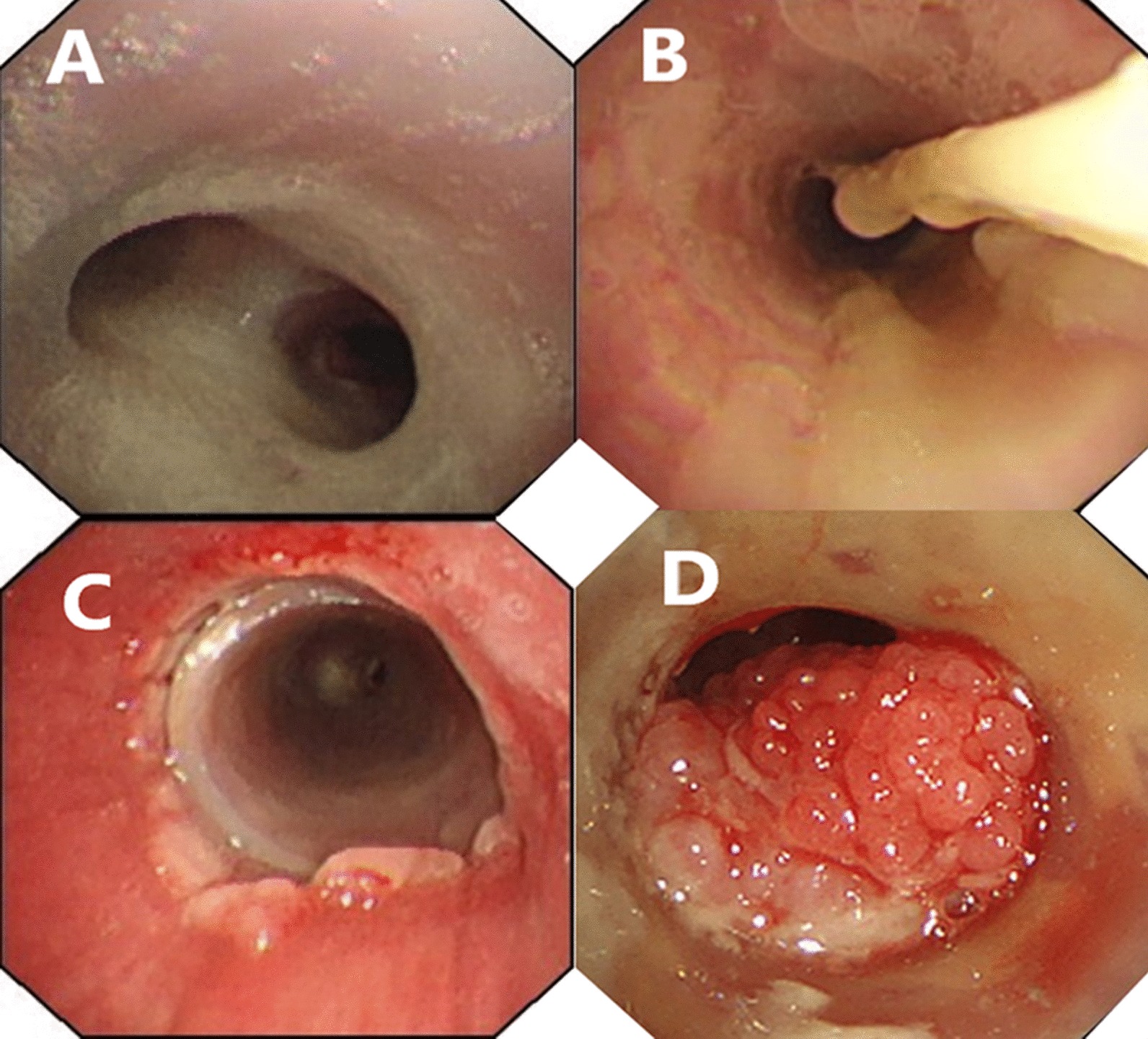
Bronchoscopic images of the complications associated with the placement of the modified Dumon stent. **a**, **b** Mucus plugging in the inner surface of the Dumon stent. **c** Mild granulomatous proliferation surrounding the stent. **d** Severe granulomatous proliferation which almost blocked the whole lumen

Stent placement was well tolerated. No airway rupture, choking, laryngeal edema, and suture dehiscence took place during follow-up. Cough and postoperative retrosternal pain were reported in almost all patients, but could be relieved after the administration of antitussive agents and analgesic scheme. All patients had various degree of respiratory infection before stenting. Exacerbation of pulmonary infection was detected in two patients (11.8%). Infection in patient No. 13 was controlled after switching to another antibiotics while patient No. 4 suffered from refractory diffuse pneumonia. Mucus plugging (Fig. 5a, b) was common after stenting, but was rated as mild and could be improved via the use of expectorants. Granulomatous proliferation (Fig. 5c, d) was identified in all cases and was successfully treated by bronchoscopic cryosurgery and argon plasma coagulation except in two patients. The stent migration was diagnosed in a single patient, which prompted the removal of the stent. The main clinical outcomes were indicated in Table 2.

**Table 2:** Clinical outcome of using the modified stent to treat BPF

Indicators	No of patients	%
Initial success	16 (16/17)	94.1
Successful stent placement	17 (17/17)	100
Immediate cessation of air leakage	16 (16/17)	94.1
Clinical success	13 (13/17)	76.5
Decreased volume of drainage (empyema)	11 (11/14)	78.6
Disappearance of residual cavity	6 (6/17)	35.3
Shrinkage of the residual cavity	10 (10/17)	58.8
Remove of the stent	7 (7/17)	41.2
For cured fistula	4 (7/17)	23.5
For granulomatous proliferation	2 (2/17)	11.8
For stent migration	1 (1/17)	5.9
Main complications	5 (5/17)	29.4
Exacerbation of pulmonary infection	2 (2/17)	11.8
Severe granulomatous proliferation	2 (2/17)	11.8
Stent migration	1 (1/17)	5.9

Three cases were lost to follow-up and eight died during follow-up. The cause of death included the progression of the underlying malignancy (n = 3), uncontrolled infection (n = 1), myocardial infarction (n = 2) and left heart failure (n = 2).

## Discussion

Our study suggested that placement of the modified silicone stent under rigid bronchoscopy could be an effective and safe option for refractory BPF. Our study has systematically explored the efficacy and safety of the modified silicone stent for BPF treatment with a larger sample size as compared with the published case series.

It should be stressed that the patients in our study failed to respond to, or were contraindicated to, many other existing treatment approaches. Most of these patients have been debilitated by the protracted airway infection. In addition, the schedule of follow-up was different individually, which was established mainly according to the general well-being and airway’s conditions. Regular check of bronchoscopy and chest CT was suggested, but data remained incomplete due to the poor adherence (follow-up visits could only be implemented when patients became symptomatic again in some patients).

Despite the advances in interventional pulmonology, BPF remains to be a severe and fatal complication of lobectomy or segmentectomy. The prognosis of BPF depends on the early diagnosis and proper management. Early diagnosis is challenging due to the insidious onset [17]. Currently, the main diagnostic techniques are CT and bronchoscopy. Chest CT is a common and useful technique that can detect peripheral BPF, which optimizes the planning of management and facilitate the follow-up [18]. Bronchoscopy can evaluate and locate the stump simultaneously. In addition, bronchoscopic treatment is also a valuable treatment strategy for BPF [19].

The management of BPF could be categorized into supportive and targeted treatments. The supportive strategy (i.e. drainage of thoracic cavity and maintenance of ventilation) is the initial treatment to avoid aspiration pneumonia and manage, if any, empyema. The targeted strategy aims to seal the fistula, with several techniques (i.e. surgery, endoscopy) being available. Although fistula might be resolved spontaneously or with appropriate supportive management, the targeted managements are required in the majority of cases [20]. Surgery (including open thoracostomy, completion pneumonectomy, thoracoplasty, suture closure with a vascularized pedicle of omentum or muscle) remains the cornerstone treatment approach. Although a high success rate of surgical management of BPF has been reported, the risk of recurrence is also high [1]. Besides, most patients with BPF cannot readily tolerate a second surgery. Compared to surgical management, the endoscopic closure has the advantages of having a lower cost, being less invasive, and having a wider scope of application. Therefore, bronchoscopic interventions might be considered as the alternative approach to surgery in some conditions [21]. Ravindra et al. [22] subdivided the bronchoscopic management into the use of sealant and occlusive devices. The reported sealants mainly included collagen matrix plugs [23], collagen screw plugs [24], different bio-glues [25, 26], and synthetic hydrogel [27]. The utility of these materials was restricted to small BPFs (< 3 mm in size). The other strategy is to apply occlusive devices, which mainly consisted of stents and the modified stents such as Amplatzar™ devices [28, 29], endobronchial valves [30], and the endobronchial Watanabe spigot [31]. However, some of these devices suffered from the limitation of being expensive or cannot routinely be available in many developing countries. Therefore, the use of stent is relatively more extensive and has been proven to be effective [10, 32, 33, 12]. Stents can occlude the segment(s) or lobar bronchus associated with BPF, which provides for a rest for parenchyma and promotes distal healing.

Recently, the use of various types of stents for fistula closure have been reported. Most of these studies were comprised of small series with limited evidence, except that Han et al. [32] reported the use of a new customized covered metallic stent in a larger study population (n = 148). Despite the high success rate, the stents used to be customized from the manufacturer. The time-consuming process has limited the clinical application especially in emergency situations. Besides, the study by Han et al. reported a manufacturing defect (a 1 mm hole in the stent bullet) and eight stent damage. Thus, the rupture of membrane and the damage of metal components might be common and could be a major drawback for covered metallic stent. The silicone stent shows some merits in terms of the durability and the ease of removal. Moreover, the smooth inner surface may have facilitated the clearance of airway secretions and the Y-shape structure and studs outside the stent have been designed to prevent the stent from migration. The major weakness of silicone stent is the limited number of types and the poor adaptability. However, the shortcoming can be overcome by modifying (cutting, suturing and/or nesting) the stent manually on site. In our study, we have modified the stent on site to generate an individualized stent without a long waiting time. As we have described above, each method has its own features and candidates, and therefore it would not be practical to compare these methods directly. Our results have nonetheless suggested that the modified stent might be more appropriate for some patients who are more refractory to conventional treatment.

There remain some disadvantages for the modified silicone stent. First, a stent acts as a foreign body, which would predispose to the proliferation of granulomatous tissues and infection. Hence, it is suggested to be removed when the fistula was cured. However, there is no consensus on the optimal time for stent removal. Second, the displacement could be potentially life-threatening despite the low incidence. Third, the stent must be deployed under rigid bronchoscopy, which require specialized equipment and well-trained team.

The successful management of BPF depends on multiple factors: appropriate selection of candidates, secure blocking the fistulous tract, sustainable elimination of all inflammatory effusion administration of sensitive antibiotics. All these should be viewed as individualized management since no single approach yielded superiority against other approaches. Moreover, placement of the modified Dumon stent cannot be viewed as palliative care because the fistula was cured in four of the patients in our study. Therefore, stent placement might also be a radical treatment modality in a subgroup of patients.

The major limitations of our study have also been recognized. This was a retrospective study performed in a single center, making it difficult to determine the generalizability of our findings. Furthermore, we cannot compare the different therapeutic options in bronchial fistula treatment, given the low prevalence of BPF and the different health condition of patients.

## Conclusions

In conclusion, the modified silicone stent is effective and safe for the treatment of post-surgical BPF, especially in patients with a large fistula size and surgery is inappropriate or contraindicated.

## Supplementary Information

**Additional file 1**. The procedure of modifying and placing the silicone stent.

**Additional file 2**. The type of the stents.

## Data Availability

The dataset used are available from the corresponding author on reasonable request.

## Abbreviations

BPF: Bronchopleural fistula
CT: Computed tomography

## References

[1] Cooper WA, Miller JJ (2001). Management of bronchopleural fistula after lobectomy. Semin Thorac Cardiovasc Surg.

[2] Sirbu H, Busch T, Aleksic I, Schreiner W, Oster O, Dalichau H (2001). Bronchopleural fistula in the surgery of non-small cell lung cancer: incidence, risk factors, and management. Ann Thorac Cardiovasc Surg.

[3] Boudaya MS, Smadhi H, Zribi H, Mohamed J, Ammar J, Mestiri T (2013). Conservative management of postoperative bronchopleural fistulas. J Thorac Cardiovasc Surg.

[4] Cerfolio RJ (2001). The incidence, etiology, and prevention of postresectional bronchopleural fistula. Semin Thorac Cardiovasc Surg.

[5] Farkas EA, Detterbeck FC (2006). Airway complications after pulmonary resection. Thorac Surg Clin.

[6] Bribriesco A, Patterson GA (2018). Management of postpneumonectomy bronchopleural fistula: from thoracoplasty to transsternal closure. Thorac Surg Clin.

[7] de la Riviere AB, Defauw JJ, Knaepen PJ, van Swieten HA, Vanderschueren RC, van den Bosch JM (1997). Transsternal closure of bronchopleural fistula after pneumonectomy. Ann Thorac Surg.

[8] Sarkar P, Chandak T, Shah R, Talwar A (2010). Diagnosis and management bronchopleural fistula. Indian J Chest Dis Allied Sci.

[9] Oki M, Saka H, Hori K (2017). Airway stenting in patients requiring intubation due to malignant airway stenosis: a 10-year experience. J Thorac Dis.

[10] Dutau H, Toutblanc B, Lamb C, Seijo L (2004). Use of the Dumon Y-stent in the management of malignant disease involving the carina: a retrospective review of 86 patients. Chest.

[11] Ryu YJ, Kim H, Yu CM, Choi JC, Kwon YS, Kim J (2006). Comparison of natural and Dumon airway stents for the management of benign tracheobronchial stenoses. Respirology.

[12] Tsukada H, Osada H (2005). Use of a modified Dumon stent for postoperative bronchopleural fistula. Ann Thorac Surg.

[13] Ferraroli GM, Testori A, Cioffi U, De Simone M, Alloisio M, Galliera M (2006). Healing of bronchopleural fistula using a modified Dumon stent: a case report. J Cardiothorac Surg.

[14] Tayama K, Eriguchi N, Futamata Y, Harada H, Yoshida A, Matsunaga A (2003). Modified Dumon stent for the treatment of a bronchopleural fistula after pneumonectomy. Ann Thorac Surg.

[15] Batra H, Yarmus L (2018). Indications and complications of rigid bronchoscopy. Expert Rev Respir Med.

[16] Zeng J, Wu X, Zhang M, Lin L, Ke M (2020). Modified silicone stent for difficult-to-treat massive hemoptysis: a pilot study of 14 cases. J Thorac Dis.

[17] Shekar K, Patel N, Chusid J, Shah R, Talwar A (2010). Bronchopleural fistula: an update for intensivists. J Crit Care.

[18] Dunne R, Colson YL, Gill RR (2014). Bronchopleural fistula and the role of contemporary imaging. J Thorac Cardiovasc Surg.

[19] Lois M, Noppen M (2005). Bronchopleural fistulas: an overview of the problem with special focus on endoscopic management. Chest.

[20] Okami J, Tokunaga T, Kanou T, Kunou H, Ishida D, Fujiwara A (2017). Randomized study comparing equal height staples with graduated height staples in bronchial closure. Ann Thorac Surg.

[21] West D, Togo A, Kirk AJ (2007). Are bronchoscopic approaches to post-pneumonectomy bronchopleural fistula an effective alternative to repeat thoracotomy?. Interact Cardiovasc Thorac Surg.

[22] Singla A, Bhat RS, Godara R, Lokanath C, Cutaia M (2018). An innovative solution for prolonged air leaks: the customized endobronchial silicone blocker. J Bronchol Interv Pulmonol.

[23] Paul S, Talbot SG, Carty M, Orgill DP, Zellos L (2007). Bronchopleural fistula repair during Clagett closure utilizing a collagen matrix plug. Ann Thorac Surg.

[24] Araki M, Sato T, Morino S, Kawanami R, Yoshitani M (2006). Bronchoscopic treatment of postpneumonectomy bronchopleural fistula with a collagen screw plug. J Thorac Cardiovasc Surg.

[25] Potaris K, Mihos P, Gakidis I (2003). Experience with an albumin-glutaraldehyde tissue adhesive in sealing air leaks after bullectomy. Heart Surg Forum.

[26] Ranu H, Gatheral T, Sheth A, Smith EE, Madden BP (2009). Successful endobronchial seal of surgical bronchopleural fistulas using BioGlue. Ann Thorac Surg.

[27] Mehta HJ, Malhotra P, Begnaud A, Penley AM, Jantz MA (2015). Treatment of alveolar-pleural fistula with endobronchial application of synthetic hydrogel. Chest.

[28] Kramer MR, Peled N, Shitrit D, Atar E, Saute M, Shlomi D (2008). Use of Amplatzer device for endobronchial closure of bronchopleural fistulas. Chest.

[29] Fruchter O, El RBA, Abdel-Rahman N, Saute M, Bruckheimer E, Kramer MR (2014). Efficacy of bronchoscopic closure of a bronchopleural fistula with amplatzer devices: long-term follow-up. Respiration.

[30] Fiorelli A, D'Andrilli A, Cascone R, Occhiati L, Anile M, Diso D (2018). Unidirectional endobronchial valves for management of persistent air-leaks: results of a multicenter study. J Thorac Dis.

[31] Maki Y, Fujikura Y, Tagami Y, Hamakawa Y, Sasaki H, Misawa K (2019). Empyema with multiple bronchopleural fistulae improved by bronchial occlusion using an endobronchial watanabe spigot with the push and slide method. Intern Med.

[32] Han X, Yin M, Li L, Zhu M, Ren K, Qi Y (2018). Customized airway stenting for bronchopleural fistula after pulmonary resection by interventional technique: single-center study of 148 consecutive patients. Surg Endosc.

[33] Cao M, Zhu Q, Wang W, Zhang TX, Jiang MZ, Zang Q (2016). Clinical application of fully covered self-expandable metal stents in the treatment of bronchial fistula. Thorac Cardiovasc Surg.

