# Supplementary material for: Modified silicone stent for the treatment of post-surgical bronchopleural fistula: a clinical observation of 17 cases
Source: BMC Pulm Med. 2021 Jan 6;21:10. doi: 10.1186/s12890-020-01372-8 (PMC7789393; doi:10.1186/s12890-020-01372-8)
Supplement: Supplementary file 1 — Additional file 1. The procedure of modifying and placing the silicone stent. [file 12890_2020_1372_MOESM1_ESM.docx]

**Online supplement**

**Modified silicone stent for the treatment of post-surgical bronchopleural fistula: A clinical observation of 17 cases**

Junli Zeng ^1^, Xuemei Wu ^1^, Zhide Chen ^1^, Meihua Zhang ^1^, Mingyao Ke ^1^*

1. Department of Respiratory Centre, The Second Affiliated Hospital of Xiamen Medical College, Xiamen, Fujian Province, China, 361000

**Methods**

*The procedure of modifying and placing the silicone stent*

The flexible bronchoscopy was performed before stent placement to identify the site of fistula orifice and clear away the secretion. For the fistula which could not be visualized by bronchoscopy, a balloon occlusion test or instillation of methylene blue would be performed. The site and size of the fistula were measured by multi-slice CT (with three-dimensional reconstructions) and bronchoscopy.  We used the sterilized straight stent segments with different diameter as a measurement tool to determine the optimal size of the stent. The sterilized straight stent segments with different outer diameters were used under rigid bronchoscopy to detect the optimal size of the stent. According to the measurement, the silicone stent (TRACHEBRONXANE™ DUMON®, Novatech, France) with the optimal diameter was chosen. The selected stent was then modified manually on site to fit the individual airways. One of the lateral branches of the Y-shaped stent was randomly selected as the occluded branch. Based on the findings of flexible bronchoscopy, the length of each branch was adjusted with scissors. The distal end of the occluded branch was sealed with nylon wires by continuous locking suture, in which the distance between the continuous sutures should be less than 2 mm (5-10 mm in common surgery) to avoid leakage. Finally, the modified silicone stent consisted of the occluded branch, the main branch and the lateral branch (**Figure. 1-A**). We fitted a suitable stent ring by nesting or suturing to the selected stent in case the size of stent was not optimal (**Figure. 1-B, C**).

Next, the modified stent was placed into the folding system. Under general anesthesia, the stent was implanted into the appropriate location through the rigid bronchoscope, and adjusted by using the surgical grasping forceps or foreign-body forceps. Finally, the modified Dumon stent was placed and the involved bronchus (lobar bronchus or main bronchus) was excluded according to the site of fistula. Because of the corresponding lobe of the bronchus that we have excluded were resected before stent placement in most of our cases, the operation would hardly result in a major decrease in the lung function. All patients were carefully and continuously monitored. Within one week after stenting, all patients underwent bronchoscopy and chest CT to identify the location of stent and airway patency.
