# Supplementary material for: Modified silicone stent for the treatment of post-surgical bronchopleural fistula: a clinical observation of 17 cases
Source: BMC Pulm Med. 2021 Jan 6;21:10. doi: 10.1186/s12890-020-01372-8 (PMC7789393; doi:10.1186/s12890-020-01372-8)
Supplement: Supplementary file 2 — Additional file 2. The type of the stents. [file 12890_2020_1372_MOESM2_ESM.docx]

**Supplementary table. The type of the stents**

| Type of stent | | Number of Patients (%) |
| --- | --- | --- |
| Stent dimension (Y a-b-b) § | |  |
|  | Y18-14-14 | 5 (29.4%) |
|  | Y16-13-13 | 5 (29.4%) |
|  | Y15-12-12 | 2(11.8%) |
|  | Y14-10-10 | 5 (29.4%) |
|  | LMB | 3 (17.6%) |
|  | RMB | 5 (29.4%) |
|  | RIB | 4 (23.5%) |
|  | RSB | 2 (11.8%) |
|  | LIB | 2 (11.8%) |
|  | LSB | 1 (5.9%) |

Abbreviation: RMB: right main bronchus; LMB: left main bronchus; right superior lobar bronchus; LSB: Left superior lobar bronchus; RSB: right superior lobar bronchus; RIB: right inferior lobar bronchus, LIB: left inferior lobar bronchus.

§: “Y” refers to the shape of stent, “a” and “b” refer to the outer diameters of the main branch and the two branches, respectively. The diameter is presented as millimeters.
